# Impact of tobacco smoking on the risk of developing 25 different cancers in the UK: a retrospective study of 422,010 patients followed for up to 30 years

**DOI:** 10.18632/oncotarget.24724

**Published:** 2018-04-03

**Authors:** Louis Jacob, Moritz Freyn, Matthias Kalder, Konstantinos Dinas, Karel Kostev

**Affiliations:** ^1^ Faculty of Medicine, University of Paris 5, Paris, France; ^2^ University Clinic, Philipps University of Marburg, Marburg, Germany; ^3^ Faculty of Medicine, Aristotle University of Thessaloniki, Thessaloníki, Greece; ^4^ Epidemiology Research, IQVIA, Frankfurt, Germany

**Keywords:** tobacco smoking, cancer, risk factor, general practices, the UK

## Abstract

**Background:**

The aim of this study was to analyze the impact of tobacco smoking on the risk of developing 25 different cancers in patients followed for up to 30 years in general practices in the UK.

**Methods:**

This study included all individuals with at least one visit to one of 196 general practitioners’ offices in the UK between January 1988 and December 2008 (index date). Only individuals with documented smoking status were included. Smokers and non-smokers were matched (1:1) by age, gender, index year, body mass index, and physician. The main outcome of the study was the risk of cancer as a function of smoking status. Data regarding a total of 25 cancers were available for the present analysis. The risk of cancer was analyzed using Cox’s regression model.

**Results:**

The present retrospective study included 211,005 smokers and 211,005 non-smokers. The mean age was 36.5 years (SD = 12.5 years) in men and 34.3 years (SD = 13.1 years) in women. There was a slightly positive association between smoking and any cancer in both men (HR = 1.07) and women (HR = 1.03). Smoking was further found to be positively associated with several cancers, such as liver cancer, bladder and kidney cancers, pancreas cancer, and lymphoma. By contrast, the use of tobacco was negatively associated with the risk of developing skin cancer, prostate cancer, multiple myeloma, endometrial carcinoma, or breast cancer.

**Conclusions:**

Smoking increased the overall risk of cancer in primary care practices in the UK. In addition, smoking was predominantly positively and less frequently negatively associated with numerous specific cancers.

## INTRODUCTION

In 2015, more than 1.1 billion individuals worldwide smoked tobacco [1]. The prevalence of adult smoking was around 16% in the UK in 2016, this number being higher in men (17.7%) than in women (14.1%) [2]. The effects of smoking on health are considerable in this country, as smoking is the leading cause of preventable death and is responsible for approximately 80,000 deaths annually [2].

Several longitudinal studies in recent years have focused on the impact of smoking on the risk of developing cancer. A 2001 study conducted in the Netherlands which included more than 1,100 patients from hospital showed that smoking was significantly positively associated with cutaneous squamous cell carcinoma, the risk being higher in current smokers than in former smokers [3]. Later, researchers from Denmark found in nearly 20,000 individuals followed for up to 31 years that a reduction by 50% in cigarette smoking led to a decrease in the subsequent risk of being diagnosed with lung cancer [4]. More recently, an European study estimated that the proportion of tobacco-related cancers attributable to cigarette smoking was around 35%, with cancers of the lung and the larynx exhibiting the highest attributable fraction [5].

Although these findings are of great importance, only few works were based on recent data from primary care practices. Furthermore, since most of the previous studies investigated either cancer in general or cancers frequently associated with cigarette smoking, such as lung tumors, little is known about the association between the use of tobacco and less frequent cancers (i.e. lymphoma, stomach cancer, and kidney cancer). Therefore, our goal was to analyze the impact of tobacco smoking on the risk of developing 25 different cancers in patients followed for up to 30 years in general practices in the UK.

## RESULTS

**Figure 1:**
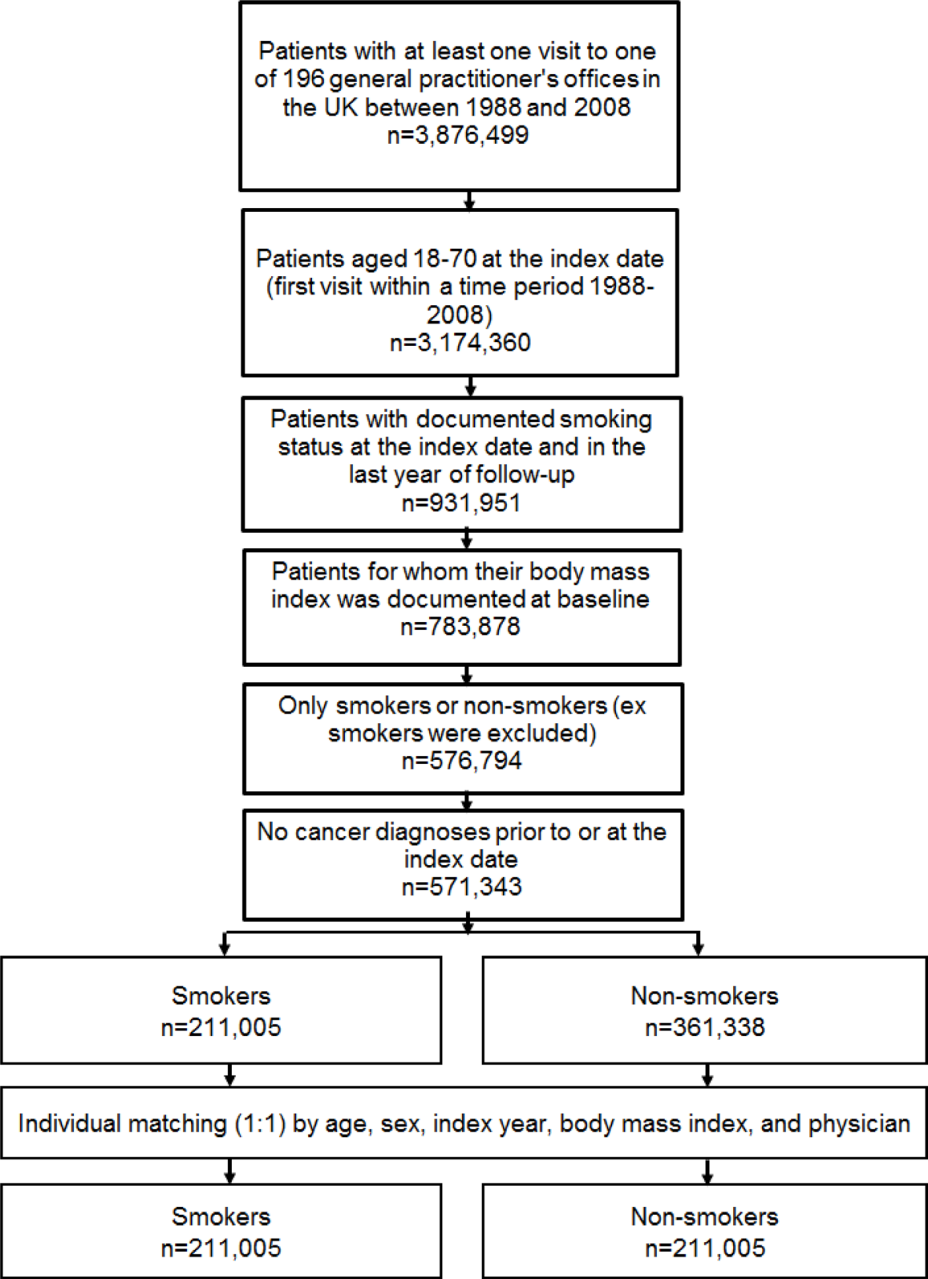
Selection of study patients

**Figure 2:**
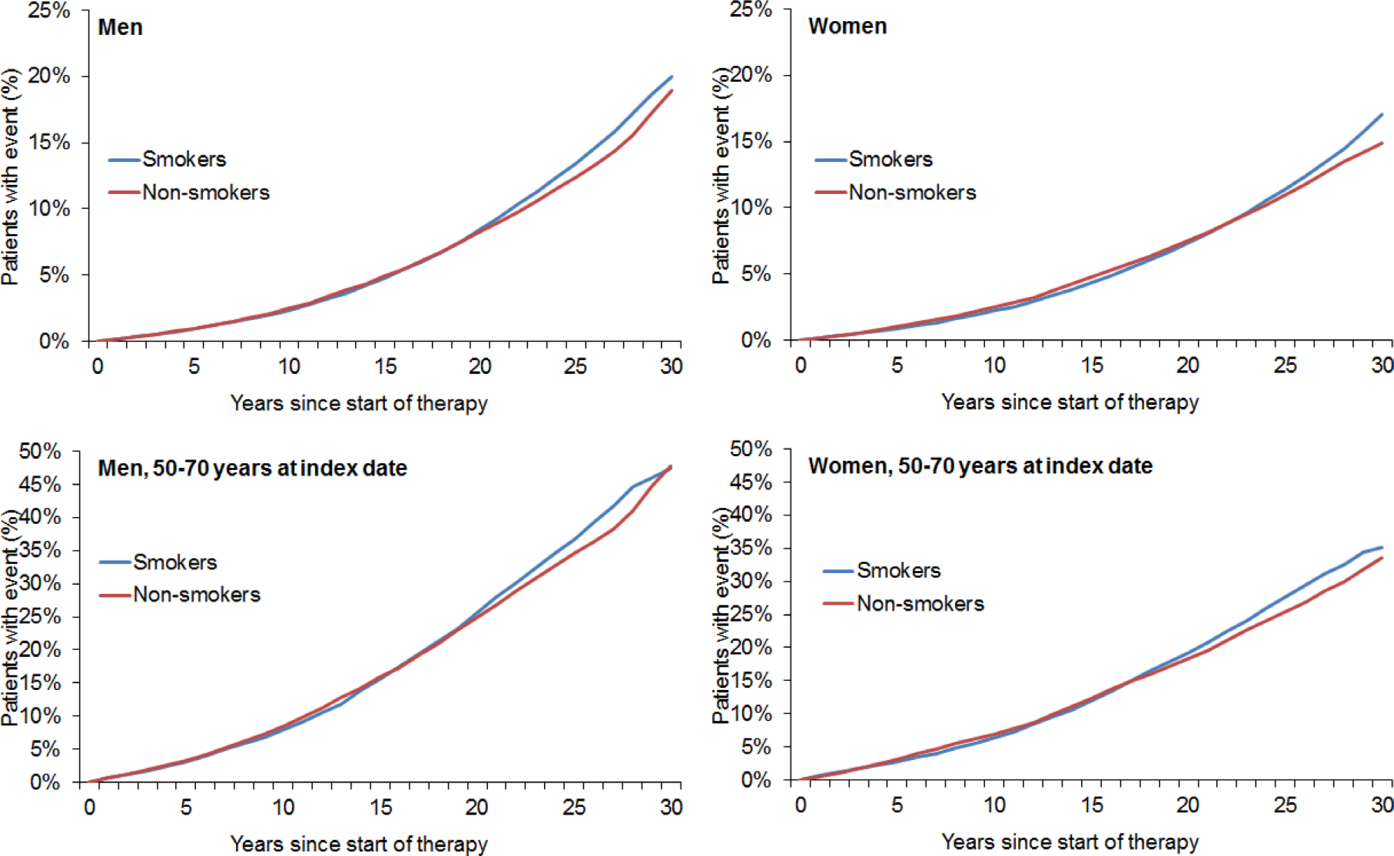
Kaplan–Meier curves for time to diagnosis of any cancer in men and women in the smoking and non-smoking groups

**Figure 3:**
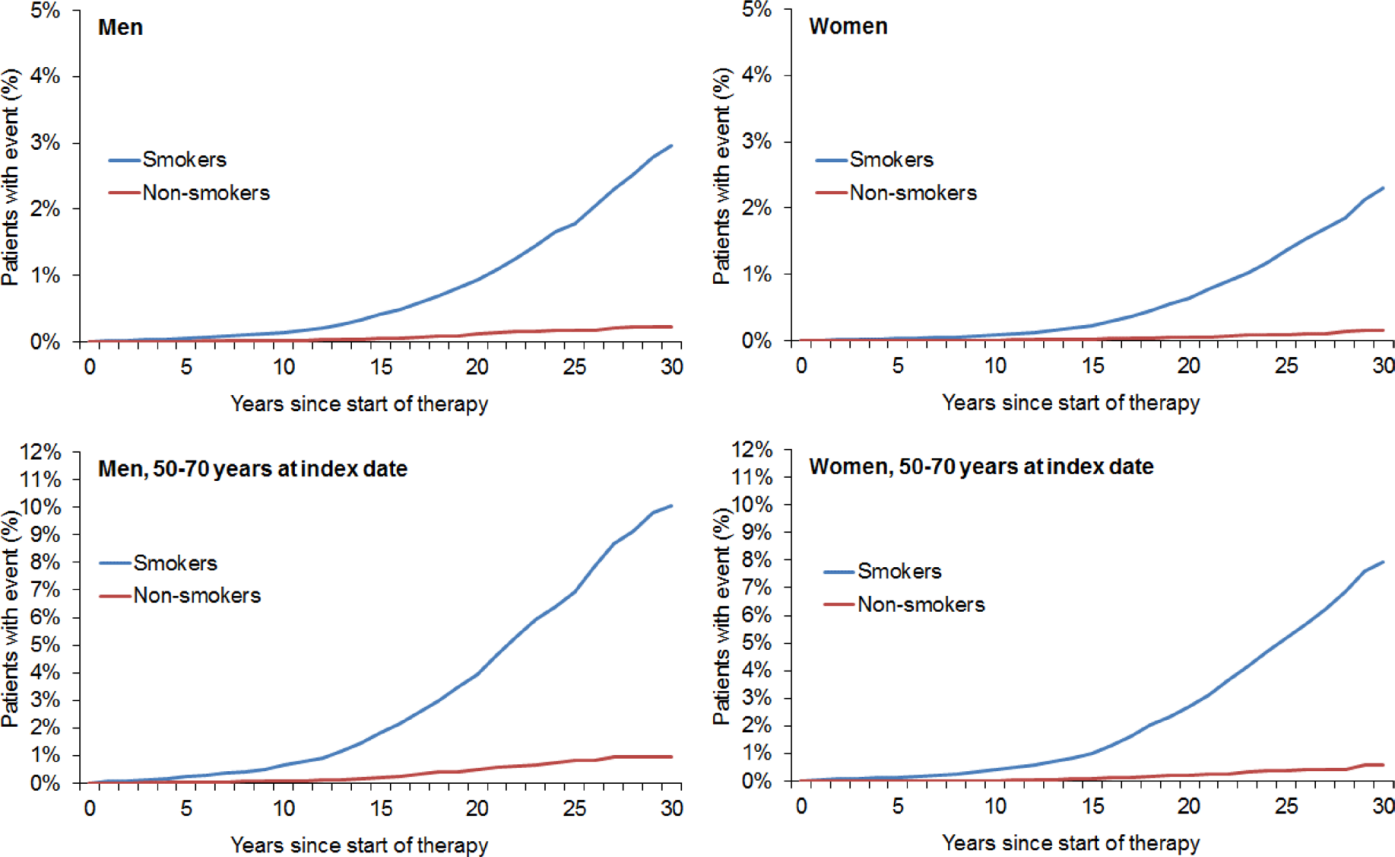
Kaplan–Meier curves for time to diagnosis of lung cancer in men and women in the smoking and non-smoking groups

After 1:1 matching, the present retrospective study included 211,005 smokers and 211,005 non-smokers (Figure 1). The mean age was 36.5 years (SD = 12.5 years) in men and 34.3 years (SD = 13.1 years) in women (Table 1). A total of 48.8% of participants were men and 51.2% were women. After 30 years of follow-up, 20.0% of male smokers, 19.0% of male non-smokers, 17.0% of female smokers, and 14.9% of female non-smokers had developed any of the included types of cancer (Figure 2). Lung cancer diagnoses were found in 2.96% of male smokers, 0.22% of male non-smokers, 2.31% of female smokers, and 0.15% of female non-smokers (Figure 3).

**Table 1:** Baseline characteristics of study patients after 1:1 matching by age, gender, body mass index, index year, and physician

Variable	Men		Women	
	Smokers (%)	Non-smokers (%)	Smokers (%)	Non-smokers (%)
Age at index date				
Age (mean, SD)	36.5 (12.5)	36.5 (12.5)	34.3 (13.1)	34.3 (13.1)
Age ≤30	38.2	38.2	48.2	48.2
Age 31–40	27.4	27.4	22.5	22.5
Age 41–50	19.3	19.3	16.1	16.1
Age 51–60	10.4	10.4	8.5	8.5
Age 61–70	4.7	4.7	4.7	4.7
BMI (mean, SD)	26.5 (4.4)	26.5 (4.4)	25.7 (5.5)	25.7 (5.5)
BMI ≤20	3.8	3.8	11.7	11.7
BMI 21–25	36.7	36.7	42.8	42.8
BMI 26–30	41.4	41.4	26.9	26.9
BMI 31–35	13.6	13.6	11.8	11.8
BMI >35	4.5	4.5	6.8	6.8

The results of the multivariate regression models conducted in men and women are displayed in Tables 2 and 3. In the overall male population, a positive association between smoking and any of the included types of cancer was found (HR = 1.07). In addition, smoking was positively associated with 11 cancers (bronchus and lung: HR = 9.92; larynx: HR = 5.17; liver: HR = 2.83; nasal cavity, middle ear, sinuses: HR = 2.61; bladder: HR = 2.32; lip, oral cavity, and pharynx: HR = 2.12; esophagus: HR = 1.97; pancreas: HR = 1.83; stomach: HR = 1.40; kidney: HR = 1.26; and lymphomas: HR = 1.24), and negatively associated with three cancers (skin: HR = 0.74; prostate: HR = 0.71; multiple myeloma: HR = 0.67).

**Table 2:** Association between smoking and the risk of cancer in men followed for up to 30 years

	All men				Men aged 50–70 at index date			
Cancer diagnosis	Proportions of cancer in smokers (%)	Proportions of cancer in non-smokers (%)	HR (95% CI)	*p*-value	Proportions of cancer in smokers (%)	Proportions of cancer in non-smokers (%)	HR (95% CI)	*p*-value
Cancer total (C00-C99)	20.0	19.0	1.07 (1.04–1.11)	<0.001	47.4	47.7	1.04 (1.00–1.09)	0.056
**Positive association**								
Bronchus and lung (C34)	2.96	0.22	9.92 (8.08–12.17)	<0.001	10.03	0.94	8.91 (6.89–11.52)	<0.001
Larynx (C32)	0.27	0.03	5.17 (3.16–8.44)	<0.001	0.78	0.09	4.69 (2.37–9.29)	<0.001
Liver (C22)	0.40	0.13	2.83 (1.85–4.32)	<0.001	1.32	0.38	2.17 (1.20–3.92)	0.011
Nasal cavity, middle ear, sinuses (C30, C31)	0.15	0.05	2.61 (1.60–4.28)	<0.001	0.64	0.26	2.47 (1.35–4.51)	0.003
Bladder (C67)	1.26	0.59	2.32 (1.98–2.73)	<0.001	5.31	3.19	2.10 (1.73–2.56)	<0.001
Lip, oral cavity, and pharynx (C00-14)	0.61	0.26	2.12 (1.71–2.63)	<0.001	0.23	0.11	2.06 (1.37–3.11)	0.001
Esophagus (C15)	0.74	0.32	1.97 (1.57–2.46)	<0.001	1.92	0.58	2.28 (1.60–3.25)	<0.001
Pancreas (C25)	0.46	0.22	1.83 (1.34–2.49)	<0.001	1.50	0.64	2.28 (1.60–3.25)	<0.001
Stomach (C16)	0.21	0.18	1.40 (1.02–1.94)	0.039	0.82	0.73	1.38 (0.93–2.05)	0.112
Kidney (C64)	0.43	0.30	1.26 (1.00–1.58)	0.048	0.80	0.87	0.83 (0.58–1.20)	0.323
Lymphomas (C81-88)	2.53	2.09	1.24 (1.13–1.36)	<0.001	6.13	5.19	1.36 (1.18–1.57)	<0.001
**Negative association**								
Skin (C43, C44)	4.78	6.98	0.74 (0.70–0.79)	<0.001	11.87	19.72	0.73 (0.67–0.79)	<0.001
Prostate (C61)	2.95	4.39	0.71 (0.65–0.76)	<0.001	8.75	15.16	0.67 (0.60–0.74)	<0.001
Multiple myeloma (C90)	0.15	0.32	0.67 (0.48–0.93)	0.018	0.54	0.67	0.59 (0.36–0.97)	0.037
**No association**								
Leukemias (C91-95)	0.51	0.46	1.00 (0.82–1.22)	0.972	1.07	1.25	0.98 (0.73–1.34)	0.920
Colon, rectum, anus (C17-C21)	1.38	2.03	0.89 (0.79–1.00)	0.051	3.91	4.99	0.80 (0.68–0.94)	0.006
Bones (C40, C41)	0.05	0.09	0.84 (0.49–1.46)	0.542	0.04	0.04	0.81 (0.22–3.02)	0.753
Testis (C62)	0.19	0.21	0.83 (0.66–1.03)	0.092	0.05	0.07	0.88 (0.32–2.43)	0.805
Thyroid gland (C73)	0.04	0.07	0.83 (0.59–1.17)	0.280	0.07	0.07	1.43 (0.46–4.52)	0.538
Brain (C71)	0.15	0.19	0.80 (0.59–1.09)	0.155	0.34	0.23	1.12 (0.64–1.95)	0.690

**Table 3:** Association between smoking and the risk of cancer in women followed for up to 30 years

	All women				Women aged 50–70 at index date			
Cancer diagnosis	Proportions of cancer in smokers (%)	Proportions of cancer in non-smokers (%)	HR (95% CI)	*p*-value	Proportions of cancer in smokers (%)	Proportions of cancer in non-smokers (%)	HR (95% CI)	*p*-value
Cancer total (C00-C99)	17.0	14.9	1.03 (1.00–1.06)	0.031	35.1	33.6	1.06 (1.01–1.11)	0.023
**Positive association**								
Bronchus and lung (C34)	2.31	0.15	14.06 (10.91–18.12)	<0.001	7.95	0.57	13.50 (9.52–19.13)	<0.001
Larynx (C32)	0.06	0.01	4.86 (2.15–10.96)	<0.001	0.12	0.01	6.72 (1.52–29.75)	0.012
Bladder (C67)	0.34	0.12	2.75 (2.07–3.64)	<0.001	0.99	0.51	2.67 (1.90–4.12)	<0.001
Esophagus (C15)	0.24	0.07	2.55 (1.73–3.77)	<0.001	0.63	0.20	2.06 (1.19–3.57)	0.010
Nasal cavity, middle ear, sinuses (C30, C31)	0.05	0.01	2.47 (1.09–5.62)	0.031	0.30	0.03	4.39 (1.25–15.43)	0.021
Vulva/vagina (C51, C52)	0.07	0.03	1.91 (1.18–3.09)	0.009	0.12	0.09	1.24 (0.53–2.86)	0.620
Cervix uteri (C53)	0.35	0.18	1.83 (1.43–2.33)	<0.001	0.37	0.13	1.59 (0.88–2.97)	0.126
Stomach (C16)	0.11	0.07	1.67 (1.08–2.57)	0.020	0.42	0.26	1.46 (0.87–2.46)	0.155
Pancreas (C25)	0.25	0.20	1.63 (1.17–2.28)	0.004	0.84	0.42	2.06 (1.19–3.57)	0.010
Lip, oral cavity, and pharynx (C00-14)	0.34	0.17	1.43 (1.12–1.83)	0.003	0.11	0.02	2.01 (1.19–3.41)	0.009
**Negative association**								
Skin (C43, C44)	3.70	5.00	0.74 (0.70–0.79)	<0.001	8.89	14.17	0.72 (0.66–0.79)	<0.001
Breast (C50)	4.04	4.22	0.92 (0.87–0.97)	0.002	7.35	6.89	0.99 (0.89–1.11)	0.913
Corpus uteri (C54)	0.21	0.38	0.60 (0.48–0.76)	<0.001	0.36	0.85	0.54 (0.37–0.79)	0.001
**No association**								
Kidney (C64)	0.23	0.15	1.27 (0.94–1.73)	0.123	0.76	0.51	1.34 (0.83–2.18)	0.232
Brain (C71)	0.17	0.12	1.22 (0.86–1.73)	0.258	0.29	0.27	1.23 (0.61–2.49)	0.568
Bones (C40, C41)	0.03	0.03	1.17 (0.61–2.23)	0.639	0.03	0.05	0.78 (0.18–3.49)	0.747
Liver (C22)	0.17	0.09	1.12 (0.73–1.74)	0.584	0.39	0.46	0.60 (0.29–1.23)	0.164
Leukemias (C91-95)	0.28	0.22	1.07 (0.83–1.37)	0.613	0.86	0.61	1.07 (0.71–1.51)	0.753
Lymphomas (C81-88)	2.14	1.74	1.02 (0.93–1.11)	0.725	3.90	4.24	0.98 (0.84–1.15)	0.829
Colon, rectum, anus (C17-C21)	0.87	0.93	1.01 (0.88–1.15)	0.938	2.37	2.86	0.93 (0.76–1.13)	0.438
Ovary (C56)	0.36	0.41	0.88 (0.74–1.06)	0.181	1.05	1.13	0.73 (0.52–1.03)	0.075
Multiple myeloma (C90)	0.14	0.13	0.86 (0.57–1.31)	0.482	0.30	0.26	1.17 (0.66–2.08)	0.590
Thyroid gland (C73)	0.11	0.13	0.62 (0.35–1.08)	0.092	0.03	0.08	0.26 (0.07–0.91)	0.036

Female smokers exhibited a 1.03-fold increase in the risk of developing any of the included types of cancer compared to non-smokers. Moreover, ten cancers (bronchus and lung: HR = 14.06; larynx: 4.86; bladder: HR = 2.75; esophagus: HR = 2.55; nasal cavity, middle ear, sinuses: HR = 2.47; vulva/vagina: HR = 1.91; cervix uteri: HR = 1.83; stomach: HR = 1.67; pancreas: HR = 1.63; and lip, oral cavity, and pharynx: HR = 1.43) were positively and three cancers (corpus uteri: HR = 0.60, skin: HR = 0.74; breast: HR = 0.92;) negatively associated with the use of tobacco.

## DISCUSSION

The present study conducted in the UK, which included more than 422,000 individuals followed for up to 30 years, showed that smoking was positively associated with 13 different types of cancers, in particular with liver cancer, bladder and kidney cancers, pancreas cancer, and lymphoma. By contrast, the use of tobacco was negatively associated with the risk of developing skin cancer, prostate cancer, multiple myeloma, cancer of corpus uteri, or breast cancer.

One interesting finding of this study using real-world data from the UK [6] is that smoking increased the risk of liver cancer in men. In a 2006 meta-analysis of 23 studies conducted in Japan, Tanaka and colleagues estimated that cigarette smoking increased the risk of primary liver cancer, although viral hepatitis and its interaction with smoking might play an important role [7]. Three years later, researchers from France found in another meta-analysis of 38 cohort studies and 58 case-control studies that current smokers displayed a 1.5-fold and former smokers a 1.1-fold increase in the risk of liver cancer when compared to never-smokers [8]. Several tobacco components (i.e. N-nitrosodimethylamine, 4-Aminobiphenyl, arsenic, and vinyl chloride) are known liver carcinogens and could explain this association [8].

We further showed that smoking was a risk factor for bladder cancer, with hazard-ratios being slightly higher in women than in men. In 2004, Quirk *et al.* observed in a U.S. population that there was a positive association between the use of tobacco and bladder cancer and that gender did not modify this association [9]. Another study with a total of more than 4.5 million years of follow-up found that the risk for this cancer was higher in former and current smokers than in never-smokers [10]. Although the prevalence of urothelial carcinoma of the bladder is low [11], these studies clearly underline the need for regular urological management and screening of smokers. In the case of kidney cancer, tobacco was found to be a significant risk factor only in men. In 2014, Washio and colleagues estimated that smoking, consumption of fatty foods, hypertension, diabetes mellitus, and obesity increased the odds of kidney cancer death in a Japanese population [12]. Interestingly, another work observed that smoking had a significant impact on renal cell carcinoma only in patients without obesity and hypertension [13]. Since our analysis was not adjusted for these variables, it is possible that they partially explain the gender difference in the association between smoking and kidney cancer.

Another major result is that smoking was positively associated with the risk of pancreatic cancer. In 2009, Brand *et al.* showed that current smokers were diagnosed with pancreas cancer six to eight years sooner than never-smokers [14]. That same year, Lynch and colleagues found in a pooled analysis including more than 3,000 participants that smoking led to a 1.8-fold increase in the risk of pancreatic cancer and that there was a positive correlation between smoking intensity and this risk [15]. The authors further underlined the fact that the odds of being diagnosed with pancreatic cancer were similar for former smokers who had stopped smoking more than 15 years ago and never-smokers. Pancreatic adenocarcinoma is one of the leading causes of cancer death in the world, and cigarette smoking seems to be one of the most consistent environmental risk factors for the development of this type of cancer. Nevertheless, screening, early detection, and treatment still pose considerable challenges. Therefore, primary care physicians need to consider this information while performing checkups in smokers.

This study also showed an association between smoking and lymphoma. In 2003, Morton *et al.* estimated in a case-control study including 1,319 patients that cigarette smoking had a significant impact on the risk of follicular lymphoma but not on the risk of all non-Hodgkin lymphoma subtypes [16]. These results were corroborated two years later in a pooled analysis, as cigarette smoking consistently increased the odds of developing follicular lymphoma [17]. The effects of tobacco on non-Hodgkin lymphoma are likely mediated via somatic mutations such as the (14;18) translocation involving the immunoglobulin heavy chain gene on chromosome 14 and the *bcl-2* gene on chromosome 18, but also via immunosuppression such as a decrease in the responsiveness of T cells. Kamper-Jørgensen and colleagues showed that there was a 1.1-fold increase in the risk of being affected by Hodgkin lymphoma in ever-smokers compared to never-smokers [18]. These findings are in line with previous research which found that the risk of Hodgkin lymphoma increased with the number of cigarette packs per day, this association being of particular importance for mixed cellularity subtypes [19]. Similarly to non-Hodgkin lymphoma, it is possible that carcinogenesis and immune dysregulations play major roles in the relation between the use of tobacco and Hodgkin lymphoma [18, 19].

Finally, in the present work, tobacco smoking had a positive impact on the odds of developing bronchus and lung cancer, larynx cancer, nasal cavity, middle ear, sinuses cancer, lip, oral cavity and pharynx cancer, esophagus cancer, and stomach cancer. These findings are in line with those of numerous previous studies [20–24].

We also showed that smoking was negatively associated with skin cancer, prostate cancer, multiple myeloma, cancer of the female genital organs, and breast cancer. These last findings must be interpreted with great caution. In a 2001 study conducted in the Netherlands, the risk of cutaneous squamous cell carcinoma was increased by tobacco smoking [3]. More recently, in a 2017 study including almost 44,000 individuals, Dusingize *et al.* found an increased risk of squamous cell carcinoma (HR = 2.3) and a decreased risk of basal cell carcinoma (HR = 0.6) in current smokers compared to never-smokers [25]. Finally, recent research suggests that previous and current smoking have a negative but non-significant impact on melanoma [26]. Since basal cell carcinomas are more frequent than squamous cell carcinoma and melanoma in the general population [27], our results could indirectly reflect the negative association between smoking and basal cell carcinoma.

A recent meta-analysis of 24 cohort studies showed that the risk of incident prostate cancer was not significantly increased in current smokers compared to never-smokers, even if results were significant when data were stratified by the amount of cigarettes [28]. In addition, another work based on biopsy data later estimated that both total and low-grade prostate cancers were not significantly associated with current and past smoking [29]. However, Rohrmann and colleagues observed in 2013 that current smokers had a reduced risk of prostate cancer compared with never-smokers [30]. Based on these contradictory findings, the negative association between smoking and prostate cancer found in the present retrospective study should be considered with caution. Since our regression analysis was not adjusted for common risk factors for the development of this cancer (i.e. ethnicity, family history, and diet), it is possible that this finding is biased. Similar precaution must be taken when analyzing the association between smoking and multiple myeloma, as recent data did not yield significant results [31].

Finally, we showed that female smokers were less likely to be diagnosed with breast and endometrial cancers than non-smokers. These findings are difficult to interpret, as other studies have found opposite results in recent decades. Although there was little evidence for a positive association between smoking and breast cancer in cohort studies published in the 1990s [32], more recent data found that smokers exhibited an increased risk for this cancer when compared to never-smokers [33]. In our work, the impact of smoking on breast cancer was not significant in women aged 50–80 years, suggesting that age might be of particular importance for this association. The importance of tobacco in the development of endometrial cancer has been the focus of several studies in different settings [34, 35]. Interestingly, a 2008 meta-analysis of 34 studies noted that endometrial cancer, which is considered as one of the most common female genital tumors, was negatively associated with ever smoking [34]. Another work published in 2014 corroborated these results, as the risk of endometrial cancer was reduced in former and current smokers when compared to never-smokers [35]. This association was not significantly impacted by tumor characteristics and any endometrial carcinoma risk factor.

This study is subject to several limitations which should be acknowledged at this point. Firstly, the assessment of cancer diagnoses relied solely on ICD codes entered by primary care physicians. However, the documentation was complete and general practitioners in the UK are usually very well informed about the health status of their patients, with many of them following patients from childhood through adulthood. Patients change physicians less frequently than in other European countries. Secondly, the mean age of patients was low at the index date, which may explain why cancer incidence was found to be low in the present analysis. This is likely due to the fact that most patients in the UK are registered in general practices for the first time as children, adolescent, or young adults. An increase in the length of follow-up might have been associated with an increase in the incidence of cancer. Such an increase was not possible, however, due to the fact that the database was only created in the late 1980s. The third limitation is that we did not have access to the number of cigarettes smoked per day. Therefore, it was not possible to estimate the impact of the number of cigarettes on the overall risk of cancer. Fourthly, as previously stated in the Discussion, no information was available regarding common sociodemographic factors (i.e. alcohol use, family history of cancer, and occupational exposition to carcinogenic substances), even though these variables are known risk factors for cancer. On the one hand, many factors should be similar for smokers and non-smokers. On the other hand, some factors, such as comorbidities experienced during the follow-up period, may also be partly traced back to smoking behavior. Finally, there were no data from hospitals and no information regarding mortality.

The main strengths of this study are the number of patients available for analysis, the length of follow-up, and the use of real-world data pertaining to diagnoses in primary care practices where diagnoses are continuously documented, allowing for unbiased exposure assessment (no recall bias).

Smoking increased the overall risk of cancer in primary care practices in the UK. In addition, smoking was predominantly positively and less frequent negatively associated with numerous specific cancers.

## METHODS

### Database

The Disease Analyzer^®^ database (IQVIA) provides information on diagnoses, prescribed treatments, laboratory values, and demographic data obtained directly and in anonymous format from the computer systems used daily in the offices of participating doctors [6]. The value of the data is monitored by means of numerous quality criteria that ensure that the database indeed provides valuable information on diagnoses (ICD-10), prescriptions (Anatomical Therapeutic Chemical classification system (ATC)), and other medical records [6]. Reviews include checks for the latest coding behavior, gapless documentation, and linkage between diagnoses and prescriptions, for example. The selection of physicians in the database complies with representativeness objectives.

### Study population

This study included all individuals with at least one visit to one of 196 general practitioners’ offices in the UK between January 1988 and December 2008. Only individuals with documented smoking status were included. The first visit in this time period was considered the index date. The inclusion criteria were as follows: (i) age between 18 and 70 years; (ii) smoking status documented at the index date and during the last year of follow-up; (iii) body mass index documented at baseline; and (iv) absence of cancer diagnosis prior to or at index date. Furthermore, since this study focused only on smokers and non-smokers, ex-smokers were excluded from the analysis. Another reason for excluding ex-smokers was missing information about the exact point in time the persons in question quit smoking. Smokers and non-smokers were matched (1:1) by age, gender, index year, body mass index, and physician (Figure 1). Participants were followed for up to 30 years.

### Study outcome and covariables

The main outcome of the study was the risk of cancer as a function of smoking status. We selected 25 cancers: bronchus and lung (ICD-10: C34), larynx (C32), liver (C22), nasal cavity, middle ear, sinuses (C30, C31), bladder (C67), lip, oral, cavity, and pharynx (C00-14), esophagus (C15), pancreas (C25), stomach (C16), kidney (C64), lymphomas (C81-88), skin (C43, C44), prostate (C61), multiple myeloma (C90), leukemias (C91-95), colon, rectum, anus (C17-C21), bones (C40, C41), testis (C62), thyroid gland (C73), brain (C71), breast (C50), vulva/vagina (C51, C52), cervix uteri (C53), corpus uteri (C54), and ovary (C56).

### Statistical analyses

Descriptive analyses were obtained for all demographic variables and mean ± standard deviations (SDs) were calculated for normally distributed variables. The cumulative incidence of any cancer or lung cancer in the smoking and non-smoking groups was calculated for up to 30 years after the index date using Kaplan-Meier curves in men and women. Cox regression models were fitted with time to first cancer diagnosis as dependent variables and smoking status as the potential predictor. Regression models were conducted separately for men and women. As the majority of patients were under 40 years old at the index date (see result section) and due to the follow-up of up to 30 years, we additionally analyzed patients aged 50–70 as a separate group.

Values of *p* < 0.05 were considered statistically significant. The analyses were carried out using SAS version 9.4.
